# The Medical, Surgical, and Hygienic Exhibition, 1900

**Published:** 1900-06-16

**Authors:** 


### THE MEDICAL, SURGICAL, AND HYGIENIC EXHIBITION, 1900.

The Medical, Surgical, and Hygienic Exhibition, which was held at the Queen's Hall, was opened on Tuesday, June 5th, and closed on Friday, June 8th. This exhibition showed evidences of increased favour among medical men and trained nurses. The attendance of the lay public appeared to have fallen off somewhat, but this afforded better facilities for investigation by professional visitors. The number of exhibitors was about the same as in previous years, the number of stalls being slightly in excess.

There appeared to be increased interest on the part of the medical practitioner in this exhibition, and he certainly had abundant material on which to bestow his attention. There was a splendid show of surgical instruments, antiseptic and other surgical dressings and appliances, and a variety of prepared foods. Drugs undoubtedly constituted a prominent feature of the exhibition, and some of the most eminent firms were represented. The interest of the nurse also was amply provided for, and the appliances, dressings, instruments, and invalid foods, which were exhibited in abundance, attracted very considerable attention.

To begin with drugs, Messrs. Parke, Davis, and Co., in their attractive display, included Standard Fluid Extracts and Cascara Sagrada, in several forms, both fluid and tablet. This firm claims to have been the first to introduce this important drug. Particular attention was directed to Mercurol, a new chemical compound of nuclein with mercury which is a powerful germicide. Their Euthymol preparations were brought into special notice. The Euthymol Powder, in patent dusting tin, should find favour with nurses. Chloretone, a new hypnotic and local anæsthetic, received marked notice from medical men. It is claimed for this product that it is very effective for gastric disorders, vomiting during pregnancy, and sea-sickness.

Messrs. Wyley's (Limited), of Coventry, had an attractive stall. The feature of this exhibit appeared to be drugs in "compressed pellet" form, "Tropels," or compressed lozenges, and also Gelatine-coated (oval) Pills, which Messrs. Wyleys claim to have first introduced into this country. Their Ophthalmic Ointment Tubes are entitled to special notice; each tube has a perforated wooden cup so shaped as to admit of direct application of the ointment to the eyelid.

Messrs. Burgoyne, Burbidges, and Co., of 12 and 16, Coleman Street, E.C., exhibited the pharmaceutical productions of Messrs. Von Heyden, of Radebeul, near Dresden. Prominence was given to Acoin, an anæsthetic which is claimed to be the only remedy for rendering sub-conjunctival injections painless. Other specialities were Collargolum, which is a metallic silver preparation soluble in water, and an effective antiseptic; and Xeroform, an odourless substitute for iodoform.

Mr. E. Merck, of Darmstadt, and 16, Jewry Street, London, E.C., exhibited a variety of drugs and pure chemicals. The chemicals of this manufacturer are undoubtedly of a high order.

Messrs. Fairchild Bros. and Foster exhibited their well-known Panopepton, and also their Pepsencia and Peptogenic Milk Powder. To distinguish the manufactures of this firm from that of others they have adopted as trade marks the words "Zymine," "Pepule," "Panopepton," "Pepsencia," and "Peptogenic."

Messrs. F. Newbery and Sons, 27 and 28, Charterhouse Square, E.C., are agents for Messrs. W. R. Warner and Co., of Philadelphia, whose productions were exhibited to a considerable extent. Mention should be made of "Ingluvin," which is the essential principle of the gizzard, and bearing the same relation to poultry that pepsin does to the higher animals. It is claimed for Ingluvin that it has effected relief where pepsin has failed and even been rejected. Another exhibit on this stall was their well-known Bromo Soda.

Messrs. Arthur and Co., 69, Berners Street, W., exhibited many of their well-known preparations, but special notice was drawn to Bromaurum, which is said to be efficacious in the treatment of rheumatism.

The Bayer Company (Limited), of Elberfeld and 19, St. Dunstan's Hill, E.C., had a well-stocked stall of their products. Prominence was given to Tannigen, an excellent specific for summer diarrhœa in children, and Protargol (proteinate of silver), a substitute for nitrate of silver. Their latest preparation, a specimen of which was exhibited, was Epicarinum Purum, a combination of naphthalin with salicylic, for the treatment of parasitic skin diseases.

There was an extensive display of drugs and preparations, antiseptic dressings, appliances, and foods on the stall of Mr. W. Martindale, 10, New Cavendish Street, W. A urine test case, compactly arranged in a small mahogany cabinet, attracted considerable notice, and a bacteriological apparatus and test case were also given prominence.

A striking exhibit was that of Messrs. Oppenheimer, Son, and Co. (Limited), 179, Queen Victoria Street, E.C., the principal features being their Palatinoids and Bi-palatinoids. Special mention should be made of their "Cerettes" (flexible jujube containers for ointments and medicated soaps), a little instrument called "The Aërizer," which is a vapouriser in contradistinction to a spray producer, and a combined Surgical and Hypodermic Instrument Case.

Messrs. Cooper and Co., 80, Gloucester Road, S.W., gave a most attractive exhibit of their pharmaceutical preparations, mineral water manufactures, and invalid foods. Their oxy-carbonated mineral waters are highly esteemed. Their "Globena" Water, which is also oxy-carbonated, is a pure sparkling table water, supplied in syphons with metal heads lined with porcelain, to prevent contact with the water. Special mention must also be made of their "Globena" Meat, Chicken, and Turtle Essences.

Messrs. Hoff, 29, New Bridge Street, E.C., had their Malt Extract on view; the Kelpion Company, 59A, Bishopsgate Street, E.C., exhibited "Kelpion," a stainless iodine ointment.

Messrs. Ingram and Royle, of 26, Upper Thames Street, E.C., the well-known agents for natural mineral waters, salts, &c., gave an extensive display, in which "Hunyadi Janos" was prominent. The Natural Sprudel Salt of Carlsbad, which is prepared from the mineral water in the form of powder, deserves particular mention, its great advantage in not being affected by variations of temperature or atmospheric exposure rendering it most reliable.

The "Orion" brand of South Australian wines came under considerable notice. The purity of these wines is guaranteed by the certificate of the Government of South Australia.

Stower's Lime Juice Cordial and Clarified Lemon Squash were exhibited by Alexander Riddle and Co., 36 and 38, Commercial Street, E.

Messrs. Maw, Son, and Thompson, of 7-12, Aldersgate Street, E.C., had an extensive display of surgical instruments and appliances, dressings, &c. The Nurse's Wallet, specially made for the district nurse, should find a ready sale. Another speciality for nurses was Kocher's Scissors, so made as to be worked by the thumb and little finger. Messrs. Weiss and Sons, of 287, Oxford Street, exhibited a most attractive array of instruments. Their new operation table, made in nickelled steel, received particular attention from the doctors. Messrs. Mayer and Meltzer, of 71, Great Portland Street, W., also exhibited in this department. Mr. W. K. Stacy, of 4, Newgate Street, E.C., among other exhibits showed a Nurse's Bag with interchangeable lining, folding wallets, which can be opened out flat for cleaning, and an aluminium wallet, which was very smart. The show of wallets was exceptionally good.

Scientific instruments were represented by Messrs. W. Watson and Sons, of 313, High Holborn, W.C. Their New Patent Dental and Surgical Lamp attracted general observation, and attention was also directed to their Clinical Thermometers. Harry W. Cox (Limited), of Cursitor Street, Chancery Lane, gave an interesting exhibition, with demonstrations, of their *x*-ray apparatus.

The exhibit of the Dowsing Radiant Heat Company (Limited), 24, Budge Row, E.C., was a feature of the exhibition, and much attention was devoted to this stall. The apparatus shown illustrated the whole system of treatment, and a fully-equipped bed was on view with the Radiant Heat in operation.

The stall of Thomas Holland, 140, South Audley Street, W., attracted considerable notice by its exhibit of the Improved Instep Arch Sock, which is an elastic appliance for use in the case of flat-foot.

The special features of the exhibit of Messrs. Anderson, Anderson, and Anderson, india rubber and waterproof manufacturers, of 37, Queen Victoria Street, E C., were their "Combination" water bed and sheet, a surgeon's operation apron (simply secured by two elastic bands), and their compact pocket douche for district nurses. It may be interesting to add that this firm equipped the "Princess of Wales" and the "Maine" hospital ships, and also the Welsh Hospital in South Africa, with water beds, waterproof sheets, &c.

(*To be continued.*)

